# ECHOPvir: A Mixture of Echinacea and Hop Extracts Endowed with Cytoprotective, Immunomodulatory and Antiviral Properties

**DOI:** 10.3390/nu15204380

**Published:** 2023-10-16

**Authors:** Ester Percaccio, Marta De Angelis, Alessandra Acquaviva, Giovanna Nicotra, Claudio Ferrante, Gabriela Mazzanti, Silvia Di Giacomo, Lucia Nencioni, Antonella Di Sotto

**Affiliations:** 1 Department of Physiology and Pharmacology “V. Erspamer”, Sapienza University of Rome, P.le Aldo Moro 5, 00185 Rome, Italy; ester.percaccio@uniroma1.it (E.P.); gabriela.mazzanti@gmail.com (G.M.); 2 Laboratory Affiliated to Istituto Pasteur Italia-Fondazione Cenci Bolognetti, Department of Public Health and Infectious Diseases, Sapienza University of Rome, P.le Aldo Moro 5, 00185 Rome, Italy; marta.deangelis@uniroma1.it (M.D.A.); lucia.nencioni@uniroma1.it (L.N.); 3 Laboratory of Virology, Department of Molecular Medicine, Sapienza University of Rome, 00185 Rome, Italy; 4 Department of Pharmacy, Botanic Garden “Giardino dei Semplici”, Università degli Studi “Gabriele d’Annunzio”, Via dei Vestini 31, 66100 Chieti, Italy; alessandra.acquaviva@unich.it (A.A.); claudio.ferrante@unich.it (C.F.); 5 EPO S.r.l., 20141 Milan, Italy; gnicotra@eposrl.com; 6 Unit of Human Nutrition and Health, Department of Food Safety, Nutrition and Veterinary Public Health, National Institute of Health, 00161 Rome, Italy; silvia.digiacomo@iss.it

**Keywords:** hop cones, viruses, immune system, antioxidant, Nrf2, influenza virus, echinacea, herbal extracts, respiratory infections, host defences

## Abstract

Respiratory viral infections continue to pose significant challenges, particularly for more susceptible and immunocompromised individuals. Nutraceutical strategies have been proposed as promising strategies to mitigate their impact and improve public health. In the present study, we developed a mixture of two hydroalcoholic extracts from the aerial parts of *Echinacea purpurea* (L.) Moench (ECP) and the cones of *Humulus lupulus* L. (HOP) that can be harnessed in the prevention and treatment of viral respiratory diseases. The ECP/HOP mixture (named ECHOPvir) was characterized for the antioxidant and cytoprotective properties in airway cells. Moreover, the immunomodulating properties of the mixture in murine macrophages against antioxidant and inflammatory stimuli and its antiviral efficacy against the PR8/H1N1 influenza virus were assayed. The modulation of the Nrf2 was also investigated as a mechanistic hypothesis. The ECP/HOP mixture showed a promising multitarget bioactivity profile, with combined cytoprotective, antioxidant, immunomodulating and antiviral activities, likely due to the peculiar phytocomplexes of both ECP and HOP, and often potentiated the effect of the single extracts. The Nrf2 activation seemed to trigger these cytoprotective properties and suggest a possible usefulness in counteracting the damage caused by different stressors, including viral infection. Further studies may strengthen the interest in this product and underpin its future nutraceutical applications.

## 1. Introduction

Respiratory infections rank as the third leading cause of mortality, with influenza accounting for 10% of these deaths [1]. Influenza is a seasonal disease caused by an enveloped virus, which typically resolves spontaneously; however, vulnerable populations, including immunocompromised individuals, children and the elderly, are at higher risk of experiencing severe outcomes [2]. Moreover, the variable efficacy of seasonal vaccines and the development of antiviral resistance to the available drugs reduce the chance of limiting the spread of influenza [3,4]. At present, there are four antiviral drugs approved by the FDA to treat influenza, including neuraminidase or polymerase inhibitors, although their use is under monitoring for the occurrence of drug resistance [4,5]. Therefore, looking at novel antiviral strategies and identifying novel targets involved in the viral life cycle are strictly needed. 

Among the possible antiviral targets, the redox-sensitive cellular pathways, especially Nrf2 (nuclear factor erythroid 2-related factor 2), have attracted great attention in recent years owing to their involvement in the control of the host defence and in the inflammatory response [6]. Indeed, the influenza virus can impair the cellular redox balance and increase viral replication by promoting oxidative stress, leading to the impairment of antioxidant defense (e.g., depletion of glutathione, GSH) [7,8,9]. 

The oxidative stress promoted by respiratory viruses, such as the recent SARS-CoV-2, leads to an inefficient immune defence and to a marked inflammatory response, which further increase the ROS release and induce excessive apoptosis in epithelial respiratory cells [6,10,11,12], thus causing severe lung injury [13], increased susceptibility to other infections [14], cardiovascular complications [15] and higher mortality risk [13]. An impaired and dysfunctional immune response is typical of influenza; in fact, it is known that alveolar macrophages are targeted and depleted in the first stage of influenza [14]. Moreover, a decrease in GSH levels, as a consequence of oxidative stress, favours Th1/Th2 balance towards Th2 [6] with a less efficient immune response [12].

In this context, redox-modulating and immunomodulating agents could represent interesting strategies to counteract the development of viral infection by boosting the host defence systems and to limit complications arising from the inflammatory response and the impaired immune response [12]. Medicinal plants represent a promising reservoir of redox-modulating agents, including polyphenols and flavonoids, thus suggesting a potential role as preventive or adjuvant strategies against viral infections [16,17,18]. 

*Humulus lupulus* L., commonly known as hop, is an officinal plant, whose cones are widely used in the brewing industry for its flavouring and preserving properties and in traditional medicine as relaxing and anxiolytic remedies [19]. Prenylated acylphloroglucinols, commonly known as bitter acids, which include α-acids (humulone derivatives) and β-acids (lupulone derivatives), as well as prenylated chalcones like xanthohumol and isoxanthohumol, represent the main compounds occurring in mature hope cones; moreover, typical volatile compounds, such as the monoterpene β-myrcene and the sesquiterpene α-humulene, have also been highlighted [20,21]. Among them, xanthohumol and prenylflavonoids have attracted great attention as bioactive natural substances for future pharmaceutical applications [22,23]. Similarly, promising healing properties, including antioxidant, antimicrobial, sedative, antiproliferative and anti-inflammatory ones, have been reported [24,25]. In our previous study, a hydroalcoholic extract from the hop cones (named HOP extract), characterized to contain 0.4% (*w*/*w*) flavonoids, was found to possess anti-influenza activity, likely related to its antioxidant power, and to restore the antioxidant defence of host cells impaired by viruses, thus underpinning further studies to better harness its antiviral power [26]. 

In line with this promising evidence and in the attempt to discover novel preventive and/or adjuvant strategies against influenza infections and their complications, in the present study, we studied a mixture of HOP and hydroalcoholic extracts from the aerial parts of *Echinacea purpurea* (L.) Moench (ECP) as a possible cytoprotective, antiviral and immunomodulatory agent. 

*E. purpurea* is a medicinal plant used traditionally as a remedy for the common cold and moderate respiratory infections, owing to its immunostimulatory, anti-inflammatory, antioxidant and antimicrobial properties [27,28,29,30]. It was found to be able to improve innate and adaptive response, particularly by stimulating macrophage phagocytosis and lymphocyte activation, and to enhance the efficacy of influenza vaccine in immunodepressed mice without triggering the release of pro-inflammatory cytokines, which are known to exacerbate respiratory viral infections [27]. Moreover, it showed anti-inflammatory properties, likely mediated by the modulation of the Nf-kB cascade [31], and virucidal activity against enveloped viruses, like the influenza virus [32,33,34]. Its benefits have been mainly ascribed to the presence of alkamides, glycoproteins, polysaccharides and polyphenols, including caffeic acid derivatives (i.e., chicoric acid, caftaric acid and chlorogenic acid) [28]. Their amount varies depending on the plant part: for instance, the amount of caftaric acid was doubled in *E. purpurea* tops with respect to roots, while C_12_ diene-diyne alkamides were mainly concentrated in the roots [35,36]. Specifically, alkylamides have been shown to act as agonists of the cannabinoid receptor 2 (CB2), and this mechanism can play a role in the immunomodulatory and anti-inflammatory properties of echinacea [29]. 

This evidence strengthens our hypothesis that combining the ECP and HOP extracts may improve the healing power of the single extracts, resulting in a blend with antioxidant, cytoprotective, anti-inflammatory, immunomodulatory and antiviral properties, which holds promise in the fight against respiratory viruses.

To perform the study, ECP and HOP extracts were characterized for their phenolic composition through spectrophotometric and chromatographic analysis. Moreover, nontoxic concentrations of ECP were mixed with HOP at the concentration that exhibited antiviral power in our previous study [26]. The extracts and their mixtures were tested in in vitro models of airway tissue (i.e., BEAS-2B and A549 cells) for their cytotoxicity/tolerability, cytoprotection towards the oxidative damage of tert-butyl hydroperoxide (tBOOH) and immunomodulatory properties in RAW 264.7 murine macrophages under stimulation with tBOOH and lipopolysaccharide. Moreover, the antiviral activity against the influenza A PR8/H1N1 virus and the modulation of the Nrf2 expression were evaluated.

## 2. Materials and Methods

### 2.1. Chemicals

All chemicals, including the Folin–Ciocâlteu phenol reagent, aluminium chloride hexahydrate (AlCl_3_ × 6 H_2_O; Ph Eur purity), quercetin (98% purity), tert-butyl hydroperoxide (tBOOH, 70% wt in H_2_O), 3-(4,5-dimethylthiazol-2-yl)-2,5-diphenyltetrazolium bromide (MTT; 98% purity), neutral red, apoptosis kit, 2,7-dichlorofluorescein diacetate, modified Griess reagent, Annexin-V-Cy3 detection kit, Triton X-100, anti-actin antibody, the solvents ethanol (EtOH; 99.5% purity) and methanol (MeOH; 99.5% purity) and the standard phenolics (>95% purity) used for the chromatographic analysis, were purchased from Merck (Darmstadt, Germany). The materials for cell cultures, including media, cofactors and antibiotics, were provided by Aurogene (Rome, Italy). The reagents for antiviral studies, if not otherwise specified, were purchased from Invitrogen (Carlsbad, CA, USA).

### 2.2. Herbal Extracts and Mixture Preparation

The hydroalcoholic dry extract from the female inflorescences of *Humulus lupulus* L. (HOP extract; code n. 3120004), standardized to contain 0.4% flavonoids, and that from the aerial parts of *Echinacea purpurea* (L.) Moench (ECP extract; code n. 3156504), containing 4% polyphenols, were kindly supplied by EPO S.r.l. (Milan, Italy). The extracts were produced using in-house extraction techniques in accordance with European Pharmacopoeia methods. To perform the study, HOP and ECP extracts were dissolved in DMSO and deionized water, respectively. Different ECP/HOP mixtures were obtained by combining a concentration of the HOP extract and the same or a halved one of ECP (1:1 and 0.5:1 ratio, respectively). The HOP concentration was selected according to previous studies [26].

### 2.3. Phytochemical Analysis

#### 2.3.1. Total Polyphenols, Tannins and Flavonoids

Total polyphenols, tannins and flavonoids were determined according to the previously standardized spectrophotometric methods described by Di Sotto et al. [37]. The total amount was quantified using the calibration curves for tannic acid (Y = 0.0391X + 0.0263; r^2^ = 0.98; polyphenols and tannins) and quercetin (Y = 0.0023X + 0.0094; r^2^ = 0.988; flavonoids) and expressed as equivalents per milligram of the extract.

#### 2.3.2. Chromatographic Analysis of the Phenolic Compounds

The phenolic pattern was investigated by using HPLC-PDA (high-performance liquid chromatography photodiode array) analysis, as previously reported [38]. Particularly, the extracts (20 μL) were dissolved in the mobile phase (1:10 dilution factor) and injected into Waters HPLC liquid chromatography (model 600 solvent pump, 2996 PDA) connected to a C18 reversed-phase column (Prodigy ODS-3, 4.6 × 150 mm, 5 μm; Phenomenex, Torrance, CA, USA). The standard phenolics, including baicalein, benzoic acid, caffeic acid, caftaric acid, carvacrol, catechin, chicoric acid, chlorogenic acid, t-cinnamic acid, p-coumaric acid, 2,3-dimethylbenzoic acid, emodin, epicatechin, t-ferulic acid, flavon, gallic acid, kaempferol, 4-hydroxybenzoic acid, 3-hydroxytyrosol, hyperoside, isoquercetin, loganic acid, naringenin, quercetin, resveratrol, rosmarinic acid, rutin, thymol, syringic acid, syringaldehyde and vanillic acid, were enclosed in the analysis. The analyses were repeated at least twice in order to ensure repeatability.

### 2.4. Cell Cultures

Human bronchial epithelial cells BEAS-2B and human lung carcinoma cells A549 were used as airway models to evaluate the cytoprotective activities of the extracts, while murine macrophages RAW 264.7 were used to assess the immunomodulatory properties of the samples. Particularly, A549 cells and RAW 264.7 macrophages were obtained from the American Type Culture Collection (ATCC), while BEAS-2B was from Merck (Darmstadt, Germany). Additionally, MDCK (Madin-Darby canine kidney) Dand cells (ATCC) were used in the antiviral studies. A549 and RAW 264.7 were cultivated in Dulbecco’s modified Eagle’s medium (DMEM), while BEAS-2B and MDCK were cultivated in RPMI 1640. The media were supplemented with 10% fetal bovine serum (FBS), 0.3 mg/mL glutamine, 100 U/mL penicillin and 100 μg/mL streptomycin. The cells were maintained at 37 °C in a 5% CO_2_ incubator and were grown according to the supplier’s instructions and previously published methods [39].

### 2.5. Treatment Schedules for the Antioxidant, Cytoprotective and Immunomodulatory Activities 

**Figure 1:**
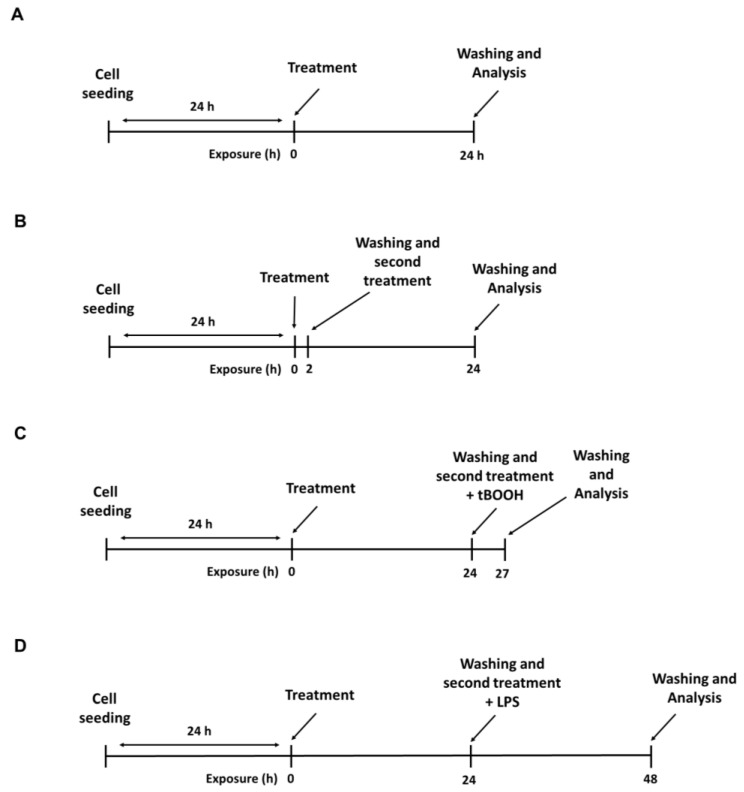
Scheduled treatment protocols applied to evaluate the cytotoxicity (**A**,**B**) and antioxidant, cytoprotective (**C**) and immunomodulatory (**D**) activities of the ECP and HOP extracts and their ECP/HOP mixtures.

The cytotoxicity of the treatments with ECP and HOP extracts (70 and/or 140 µg/mL) alone and their mixtures (i.e., ECP/HOP 70/140 µg/mL and 140/140 µg/mL) was evaluated under two different treatment protocols. In the first one, the cells were treated for 24 h (Figure 1A), while in the second one, they underwent two repeated exposures: a first exposure of 2 h followed by a second 24 h exposure (Figure 1B). The last protocol mimics the cell exposure in the antiviral assays. The antioxidant, cytoprotective activity was assessed by treating the cells with the tested samples for 24 h and then with a subtoxic concentration of the pro-oxidant agent tert-butyl hydroperoxide (tBOOH; 500 µM) for 3 h (Figure 1C). At last, the immunomodulatory properties were evaluated by treating macrophages with the tested extracts and their mixtures for 24 h and then with *E. coli* 0111:B4 lipopolysaccharide (LPS; 2 µg/mL) for another 24 h (Figure 1D). 

### 2.6. Trypan Blue Exclusion Assay

After harvesting confluent cells, a cell suspension was stained with trypan blue and then visually examined under light microscopy by using a haemocytometer to distinguish between cells including or excluding the stain: nonviable cells displayed a blue cytoplasm, while the viable ones had a clear cytoplasm. After cell counting, the total number of viable cells was measured, as previously reported [40,41].

### 2.7. Cytotoxicity Assay

To evaluate the cytotoxicity, 2 × 10^4^ cells were grown in each well of a 96-well microplate for 24 h and then exposed to the ECP and HOP extracts and their mixtures. A maximum 1% *v*/*v* DMSO in cell medium was used to avoid any solvent-related toxicity. Cytotoxicity was measured by using the 3-[4,5-dimethylthiazol-2-yl]-2,5-diphenyl tetrazolium bromide (MTT) assay using an Epoch Microplate Spectrophotometer (BioTek, AHSI, Milan, Italy) [39]. A more than 30% reduction in cell viability with respect to the control was considered a significant cytotoxic effect [42]. 

### 2.8. Cytoprotection towards the Oxidative Damage Induced by Tert-Butyl Hydroperoxide (tBOOH)

Confluent cells (2 × 10^4^ cells) were grown in 96-well microplates for 24 h and then exposed to the ECP and HOP extracts, their mixtures and a subtoxic concentration of tBOOH (500 µM). This concentration was selected in preliminary cytotoxicity studies, being able to induce about a 40–50% inhibition of cell viability. At the end of treatment, the cell viability was determined by using the MTT assay, as previously described; moreover, the intracellular levels of reactive oxygen species (ROS), as an oxidative stress parameter, and apoptosis extent were measured as follows. 

### 2.9. Intracellular Reactive Oxygen Species (ROS) Determination

To perform the assay, 2.5 × 10^5^ cells/well were grown in 24-well plates for 24 h, then treated with the extracts or their mixtures and exposed to tBOOH. At the end of the treatments, the ROS levels were measured in the cellular pellets by using the 2,7-dichlorofluorescein diacetate assay (DCFH-DA) (excitation/emission 485/528 nm) using a BD Accuri™ C6 flow cytometer (BD Biosciences, Milan, Italy) [43]. For all treatments, the mean DCF fluorescence of 50,000 cells was measured using BD Accuri^TM^ C6 software version 1.0.264.21 (BD Biosciences, Milan, Italy). An oxidation index was calculated by using the ratio of DCF fluorescence of the sample with respect to the vehicle control. 

### 2.10. Apoptosis Detection

Apoptosis was detected through flow cytometry, using Annexin-V-Cy3 staining, according to previous methods [43]. To perform the assay, 2.5 × 10^5^ cells were grown in 24-well plates and treated with the tested samples; thereafter, the pellets were collected and resuspended in PBS with Annexin-V-Cy3 fluorochrome (4 μg/mL in cell suspension). The nonfluorescent carboxyfluorescein diacetate (CFDA; 2 μg/mL) probe was added to detect viable cells; indeed, CFDA is hydrolysed by esterases into viable cells to the fluorescent metabolite carboxyfluorescein (CF), which can be measured through flow cytometry. For each sample, the mean fluorescence of CF (excitation/emission at 492/514 nm) and Annexin-V-Cy3 (excitation/emission at 543/570 nm) in 50,000 cells was determined by using a BD Accuri^TM^ C6 flow cytometer (BD Biosciences, Milan, Italy) at FL-1 and FL-2 channels, respectively. Multiparameter analysis and gating of forward and side scatter as well as fluorescence detection were performed using BD Accuri^TM^ C6 software version 1.0.264.21 (BD Biosciences, Milan, Italy) [43]. Apoptotic cells were identified by their typical forward- and side scatter (FSC-SSC) pattern (increased SSC and decreased FSC) with respect to the viable cells. 

### 2.11. Neutral Red Uptake Assay

The neutral red uptake assay was performed to evaluate the immunomodulatory abilities of the tested samples in RAW 264.7 murine macrophages according to Wu et al. with minor changes [44]. Briefly, 2 × 10^4^ cells/well were grown in 96-well plates for 24 h and treated with the tested extracts and their mixtures. After incubation, the treatments were replaced by a neutral red solution in the cell medium (50 µg/mL), and the cells were further incubated for 30 min and then washed with and lysed by an ethanol and acetic acid (1:1) solution. When the lysis was completed, the optical density was measured at 550 nm using an Epoch Microplate Spectrophotometer (BioTek, AHSI, Milan, Italy). 

### 2.12. Determination of the Secreted Nitric Oxide (NO) Levels in the Cell-Free Supernatant 

To perform the assay, RAW 264.7 murine macrophages were grown in 24-well plates (2.5 × 10^5^ cells/well). Afterwards, cells were treated with the extracts and their mixtures for 24 h and then exposed to *E. coli* 0111:B4 lipopolysaccharide (LPS; 2 µg/mL) for another 24 h. The cell supernatants were collected and centrifuged, and nitric oxide was detected by using the Griess colorimetric assay according to previously published methods [45]. Briefly, 100 µL of the cell-free supernatant was added to an equal volume of 1x modified Griess reagent (1% sulfanilamide and 0.1% N-(1-naphthyl) ethylenediamine dihydrochloride in 2.5% phosphoric acid) in ultrapure water, which was incubated in the dark for 15 min, and then the absorbance was determined at 540 nm by using an Epoch Microplate Spectrophotometer (BioTek, AHSI, Milan, Italy). The nitrite content in the samples was determined by the sodium nitrite calibration curve (10–1000 ng/mL) and expressed as a percentage of the control. 

### 2.13. Viral Infection and Titration

Confluent monolayers of BEAS-2B and A549 cells were challenged with the influenza virus A/Puerto Rico/8/34 H1N1 (PR8/H1N1 virus) strain. After the viral adsorption, the cells were washed with phosphate-buffered saline (PBS) and then incubated with the medium supplemented with 2% FBS for 24 h. Virus production was determined in the supernatants of the infected cells by measuring the hemagglutinating units (HAU) [26].

#### 2.13.1. Antiviral Activity 

The antiviral activity of the ECP and HOP extracts (70 and/or 140 µg/mL) and their mixtures (i.e., ECP/HOP 70/140 µg/mL and 140/140 µg/mL) was evaluated in both BEAS-2B and A549 cells against the infections induced by influenza A virus strain H1N1 (PR8), according to previous studies [26]. Particularly, 2.5 × 10^5^ cells/well were grown in 12-well plates for 24 h and then exposed to the tested samples under the before, during and post-infection (b.d.p.i.) protocol, in which the cells were treated 1 h before the infection, during the 1 h of viral adsorption period and 24 h post-infection: this protocol was chosen based on the results of our previous study [26]. Thereafter, supernatants and cell pellets underwent analysis. Control cells were treated with the highest concentration of the solvents in the medium. 

#### 2.13.2. Immunoblotting Analysis 

Immunoblotting analysis of the viral proteins, i.e., haemagglutinin (HA), nucleoprotein (NP) and matrix protein (M1), and of Nrf2 (nuclear factor erythroid 2-related factor 2) was carried out as previously reported [8,9]. The infected and treated cells were lysed and analysed by using SDS-PAGE followed by Western blotting using suitable anti-influenza (Merck Millipore, Darmstadt, Germany), anti-Nrf2 (Cell Signaling Technologies, Danvers, MA, USA) and anti-actin (Sigma Aldrich, St. Louis, MO, USA) antibodies. Secondary HRP-linked anti-goat and anti-mouse (Jackson ImmunoResearch, Newmarket, UK) antibodies were used as well. The membranes were developed using a Clarity Western ECL substrate (Bio-Rad, Hercules, CA, USA). 

#### 2.13.3. In-Cell Western (ICW) Assay

The ICW assay was performed using the Odyssey Imaging System (LI-COR, Lincoln, NE, USA). Madin-Darby Canine kidney MDCK cells were seeded in 96-well plates (2 × 10^4^ cells/well) and grown for 24 h. Then, cells were infected with the supernatants of BEAS-2B and A549 cells infected and treated as previously described. After 1 h, supernatants were removed, and cells were incubated for 24 h with a fresh medium supplemented with 2% FBS. After 24 h, the cells were fixed with 4% formaldehyde, washed, permeabilized with 0.1% Triton X-100 and incubated with the Odyssey blocking buffer (LI-COR Biosciences, Lincoln, NE, USA). The cells were then stained at 4 °C overnight with mouse anti-HA (1:5400; Santa Cruz Biotechnology, Dallas, TX, USA) together with a cell tag (1:2000; LI-COR Biosciences, Lincoln, NE, USA) in the Odyssey blocking buffer. Cells were then washed and stained with goat anti-mouse IRDye^TM^ 800 antibodies (1:3000; LI-COR Biosciences, Lincoln, NE, USA). Protein expression was quantified using the Odyssey Imaging System. For statistical analysis, integrated intensities of fluorescence in wells were determined using software provided with the imager station (LI-COR Biosciences, Lincoln, NE, USA). The relative amount of HA protein was obtained by normalizing the cell tag in all experiments [46]. 

### 2.14. Statistical Analysis

Statistical analysis was performed by using GraphPad Prism™ (Version 6.00) software (GraphPad Software, Inc., San Diego, CA, USA). The obtained results were expressed as the mean ± standard error of at least two experiments with at least three technical replicates per each concentration. Significant differences between the treatments were evaluated by using the one-way analysis of variance (one-way ANOVA), followed by Dunnett’s multiple comparison post-test. A *p* value < 0.05 was considered significant.

## 3. Results

### 3.1. Phytochemical Analysis

In the spectrophotometric analysis, HOP was shown to contain total polyphenol and tannin amounts almost 1.5- and 2-fold higher than ECP despite similar levels of flavonoids (Table 1). The chromatographic analysis highlighted a richer composition of phenolic compounds in both HOP and ECP extracts (Table 2). Particularly, ECP contained marked levels of caftaric acid, chicoric acid and 3-hydroxytyrosol, followed by epicatechin, caffeic acid, catechin and ferulic acid, while the main compounds of HOP were 2,3-dimethylbenzoic acid and thymol, followed by isoquercetin, t-cinnamic acid, 3-hydroxytyrosol and catechin. Other phenolic acids, such as benzoic, coumaric, gallic and ferulic acids, and flavonoids, such as kaempferol, were detected but in lower amounts. 

### 3.2. Cytotoxicity of HOP and ECP Extracts and Their Mixtures in Bronchial Epithelial BEAS-2B Cells and in Lung Adenocarcinoma A549 Cells

**Figure 2:**
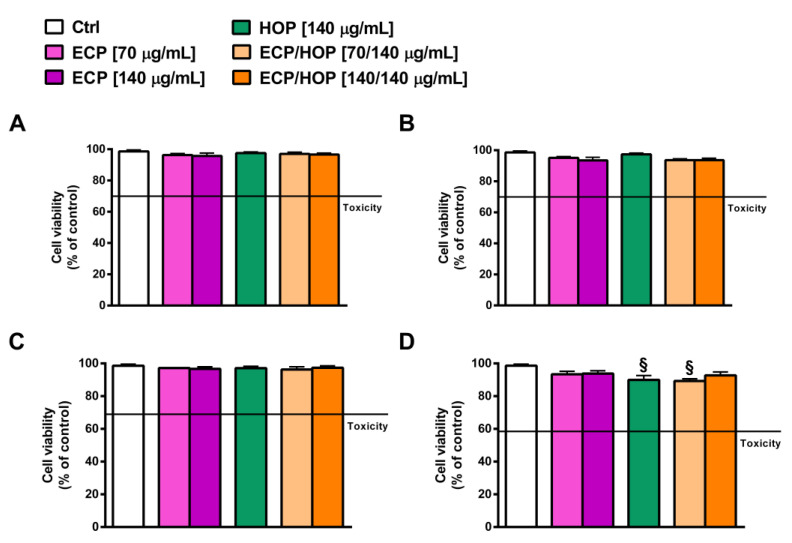
Effect of ECP (70 and 140 µg/mL), HOP (140 µg/mL) and their mixtures (70/140 and 140/140 µg/mL) on the viability of epithelial bronchial BEAS-2B and lung adenocarcinoma A549 cells after 24 h exposure (**A**,**C**) and after a 2 h treatment followed by a second 24 h exposure (**B**,**D**). Data are reported as the mean ± SEM (*n* = 9). ^§^ *p* < 0.05 vs. Ctrl. (ANOVA followed by Dunnett’s Multiple Comparison Post-Test).

Preliminary cytotoxicity studies were performed in order to avoid any effect of the HOP and/or ECP extracts and their mixtures at the selected concentrations on airway BEAS-2B and A549 cells. As explained in paragraph 2.2, the ECP/HOP mixtures were designed based on the results obtained in our previous study [26], in which a hydroalcoholic extract from the cones of *H. lupulus* exhibited antiviral properties at the concentration of 140 µg/mL. Consequently, for the present study, we chose the same concentration for the HOP extract and two concentrations for the ECP extract, namely 70 µg/mL and 140 µg/mL, resulting in two ECP/HOP mixtures at the extract ratios of 0.5:1 (70/140 µg/mL) and 1:1 (140/140 µg/mL). As expected, under our experimental conditions, neither the extracts nor their mixtures affected the viability of BEAS-2B and A549 cells after 24 h exposure (Figure 2A,C) or after an exposure of 2 h followed by a second one of 24 h (Figure 2B,D). The lack of cytotoxicity confirms that the tested samples can be further studied for their cytoprotective, immunomodulatory and antiviral properties.

**Figure 3:**
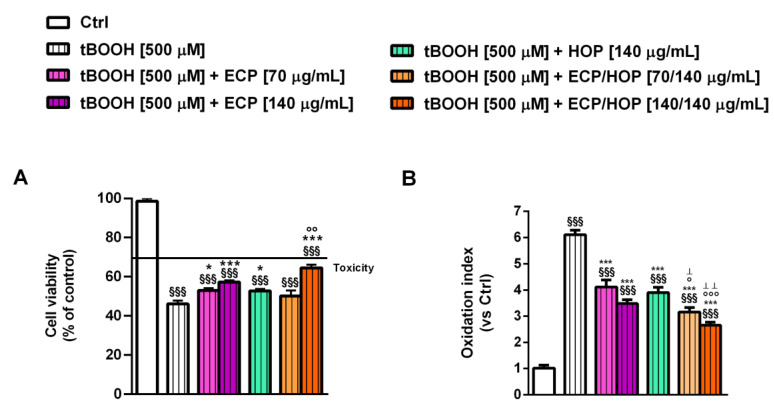
Cytoprotective effect of ECP (70 and 140 µg/mL), HOP (140 µg/mL) and their mixtures (70/140 and 140/140 µg/mL) against the oxidative damage induced by tert-butyl hydroperoxide (tBOOH) in bronchial epithelial BEAS-2B cells. The cells were treated with the extracts and their mixtures for 24 h and then with tBOOH for another 3 h. Data are displayed as the mean ± SEM (*n* = 9). (**A**) Cell viability. (**B**) Intracellular levels of reactive oxygen species (ROS). ^§§§^ *p* < 0.001, significant difference with respect to Ctrl. * *p* < 0.05 and *** *p* < 0.001 vs. tBOOH. ° *p* < 0.05, °° *p* < 0.01 and °°° *p* < 0.001, significant difference in the ECP/HOP mixture with respect to HOP extract. ^Ʇ^ *p* < 0.05 and ^ꞱꞱ^ *p* < 0.01, significant difference in the ECP/HOP mixture with respect to ECP extract. Statistical analysis performed using ANOVA followed by Dunnett’s Multiple Comparison Post-Test.

In the cytoprotective studies against oxidative damage induced by tBOOH in bronchial epithelial BEAS-2B cells, both the ECP and HOP extracts, as well as their mixture, demonstrated the ability to restore the cell viability impaired by tBOOH. The 140/140 µg/mL mixture showed a slightly but significantly higher effect compared to both HOP and ECP alone (Figure 3A). Specifically, tBOOH caused a 56% reduction in cell viability, while the ECP, HOP and their 140/140 µg/mL ECP/HOP mixture produced about a 22%, 13% and 30% cytoprotective effect, respectively (Figure 3A). Similarly, the tBOOH increased the intracellular ROS levels by approximately 6-fold with respect to the control, while the 70/140 µg/mL and 140/140 µg/mL ECP/HOP mixtures were shown to halve the levels induced by tBOOH, exhibiting antioxidant effects higher than those of the extracts. Specifically, 70 and 140 µg/mL ECP and 140 µg/mL HOP resulted in a ROS level reduction from 1.5- to 1.7-fold (Figure 3B). Consequently, the mixtures potentiated the cytoprotective effects of the extracts by 1.3–1.4 fold.

**Figure 4:**
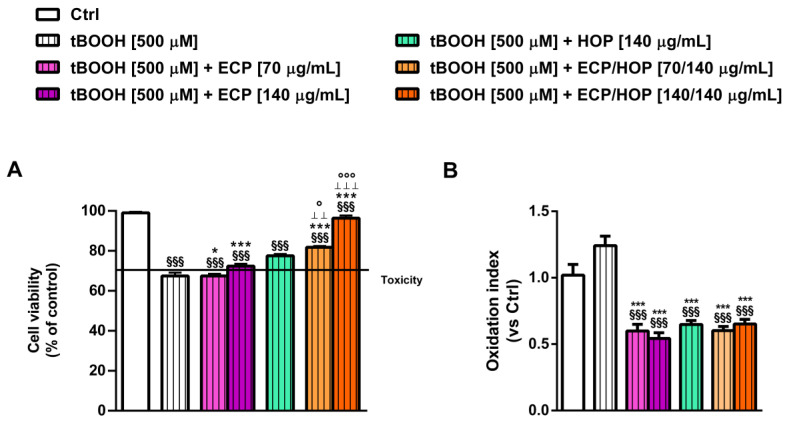
Cytoprotective effect of ECP (70 and 140 µg/mL), HOP (140 µg/mL) and their mixtures (70/140 and 140/140 µg/mL) against the oxidative damage induced by tert-butyl hydroperoxide (tBOOH) in lung adenocarcinoma A549 cells. The cells were treated with the extracts and their mixtures for 24 h and then with tBOOH for another 3 h. Data are displayed as the mean ± SEM (*n* = 9). (**A**) Cell viability. (**B**) Intracellular levels of reactive oxygen species (ROS). ^§§§^ *p* < 0.001, significant difference with respect to Ctrl. * *p* < 0.05 and *** *p* < 0.001 vs. tBOOH. ° *p* < 0.05 and °°° *p* < 0.001, significant difference in the ECP/HOP mixture with respect to HOP extract. ^ꞱꞱ^ *p* < 0.01 and ^ꞱꞱꞱ^ *p* < 0.001, significant difference in the ECP/HOP mixture with respect to ECP extract. Statistical analysis performed using ANOVA followed by Dunnett’s Multiple Comparison Post-Test.

Both the extracts and their mixtures exhibited similar effects against the tBOOH-induced damage in lung adenocarcinoma A549 cells, although with a lower efficacy of ECP compared to HOP (Figure 4A,B). This airway model was less susceptible to tBOOH-induced oxidative damage, resulting in a 33% cell viability reduction without affecting the intracellular ROS levels (Figure 4A,B). This effect could be due to the overexpression of the antioxidant defences in A549 cells, which increases the cell resilience to oxidative stress [47]. Nevertheless, both the 70/140 µg/mL and 140/140 µg/mL ECP/HOP mixtures restored the tBOOH-impaired cell viability by 1.2- and 1.4-fold, respectively. Conversely, ECP produced null or weak cytoprotection, while HOP induced a 1.3-fold increase in cell viability with respect to tBOOH (Figure 4A,B). The intracellular ROS levels were significantly lowered by both the ECP and HOP extracts as well as their mixtures (about a 33%, 40% and 45% reduction with respect to tBOOH, respectively), although with a slight but not significant potentiation of the mixtures compared to the extracts.

### 3.3. Effect of the Treatments on the Apoptosis Rate in Bronchial Epithelial BEAS-2B Cells 

**Figure 5:**
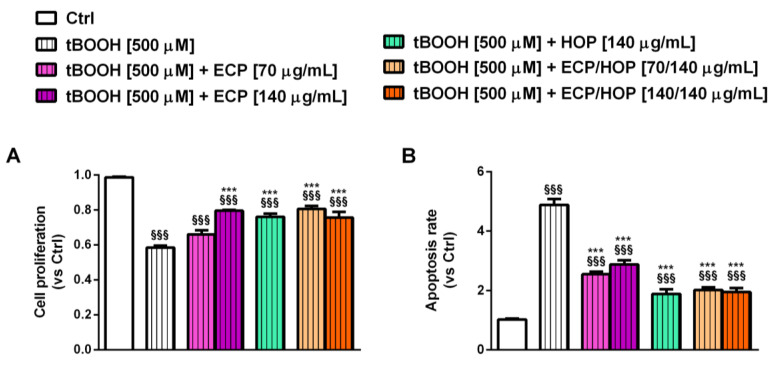
Effect of ECP (70 and 140 µg/mL), HOP (140 µg/mL) and their mixtures (70/140 and 140/140 µg/mL) on BEAS-2B cell proliferation (**A**) and apoptosis rate (**B**) impaired by the oxidative damage of tert-butyl hydroperoxide (tBOOH). The cells were treated with the extracts and their mixtures for 24 h and then with tBOOH for another 3 h. Data are displayed as the mean ± SEM (*n* = 9). ^§§§^ *p* < 0.001, significant difference with respect to Ctrl. *** *p* < 0.001 vs. tBOOH. Statistical analysis performed using ANOVA followed by Dunnett’s Multiple Comparison Post-Test.

Previous evidence highlighted that oxidative stress to airway cells, especially to bronchial epithelial cells, may induce cell death, thus leading to an impairment of their protective function; therefore, counteracting these processes may represent a suitable strategy to prevent the development of oxidative stress-induced respiratory diseases [48]. In line with this evidence and based on our results highlighting a higher susceptibility of BEAS-2B cells to the oxidative damage induced by tBOOH, in the present study, we also evaluated the effects of ECP and HOP extracts, as well as of their mixtures, on the cell proliferation and apoptosis rate as possible mechanisms of their cytoprotective properties. Under our experimental conditions, tBOOH increased the apoptosis rate by almost 5-fold with respect to the control and reduced the cell proliferation abilities by 1.7-fold (Figure 5A,B). These findings confirm the marked susceptibility of BEAS-2B cells to tBOOH damage, which in turn can activate apoptotic cell death. 

Despite the damage of tBOOH, the ECP/HOP mixtures, HOP and ECP at 140 µg/mL increased the cell proliferation by about 1.3-fold with respect to tBOOH, while ECP at 70 µg/mL exhibited no protective effects (Figure 5A). Moreover, both the extracts and their mixtures reduced the apoptotic rate from 1.7- to 2.7-fold (Figure 5B). These findings suggest that the extracts and their mixtures are able to counteract the oxidative damage of tBOOH, thus restoring the BEAS-2B cell proliferation abilities and blocking apoptotic cell death. This strengthens our previous results about the cytoprotective properties of the ECP/HOP mixtures. 

### 3.4. Immunomodulatory Activity in Murine Macrophages RAW 264.7

**Figure 6:**
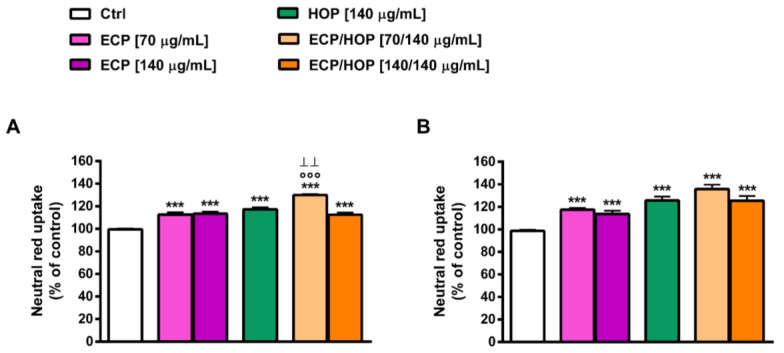
Effect of ECP (70 and 140 µg/mL), HOP (140 µg/mL) and their mixtures (70/140 and 140/140 µg/mL) on the neutral red uptake in murine macrophage RAW 264.7 cells. Cells were treated for 24 h (**A**) and for 2 h followed by a further 24 h exposure (**B**). Data are reported as the mean ± SEM (*n* = 9). *** *p* < 0.001, significant difference with respect to Ctrl. °°° *p* < 0.001, significant difference in the ECP/HOP mixture with respect to HOP extract. ^ꞱꞱ^ *p* < 0.01, significant difference in the ECP/HOP mixture with respect to ECP extract. Statistical analysis performed using ANOVA followed by Dunnett’s Multiple Comparison Post-Test.

**Figure 7:**
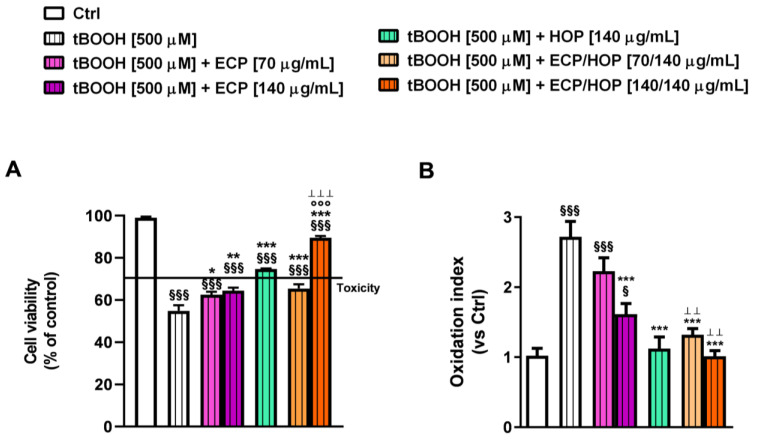
Cytoprotective effect of ECP (70 and 140 µg/mL), HOP (140 µg/mL) and their mixtures (70/140 and 140/140 µg/mL) against the oxidative damage induced by tert-butyl hydroperoxide (tBOOH) in RAW 264.7 cells. The cells were treated with the extracts and their mixtures for 24 h and then with tBOOH for another 3 h. Data are displayed as the mean ± SEM (*n* = 9). (**A**) Cell viability. (**B**) Intracellular levels of reactive oxygen species (ROS). ^§^ *p* < 0.05 and ^§§§^ *p* < 0.001, significant difference with respect to Ctrl. * *p* < 0.05, ** *p* < 0.01 and *** *p* < 0.001 vs. tBOOH. °°° *p* < 0.001, significant difference in the ECP/HOP mixture with respect to HOP extract. ^ꞱꞱ^ *p* < 0.01 and ^ꞱꞱꞱ^ *p* < 0.001, significant difference in the ECP/HOP mixture with respect to ECP extract. Statistical analysis performed using ANOVA followed by Dunnett’s Multiple Comparison Post-Test.

**Figure 8:**
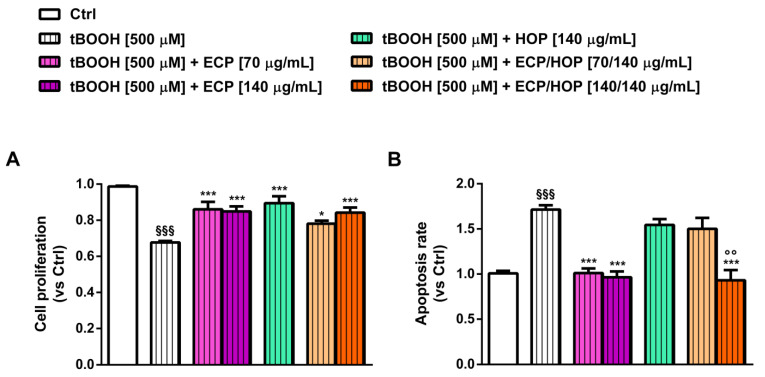
Effect of ECP (70 and 140 µg/mL), HOP (140 µg/mL) and their mixtures (70/140 and 140/140 µg/mL) on cell proliferation (**A**) and apoptosis rate (**B**) impaired by tert-butyl hydroperoxide (tBOOH) in murine macrophages RAW 264.7. Cells were pre-treated (24 h) and co-treated with tBOOH (3 h). Data are reported as the mean ± SEM (*n* = 9). ^§§§^ *p* < 0.001, significant difference with respect to Ctrl. * *p* < 0.05 and *** *p* < 0.001, significant difference with respect to tBOOH. °° *p* < 0.01, significant difference in the ECP/HOP mixture with respect to HOP extract. Statistical analysis performed using ANOVA followed by Dunnett’s Multiple Comparison Post-Test.

**Figure 9:**
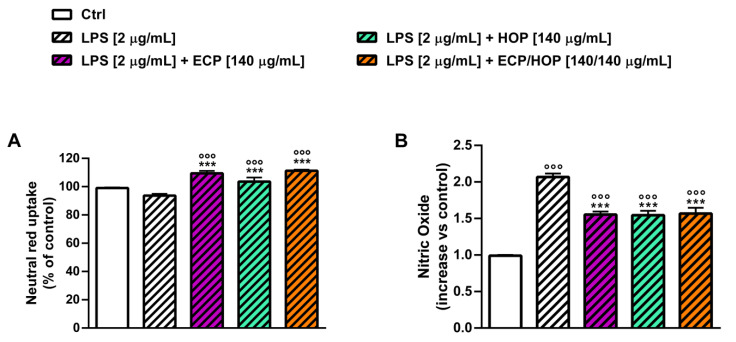
Effect of ECP (70 and 140 µg/mL), HOP (140 µg/mL) and their mixtures (70/140 and 140/140 µg/mL) on neutral red uptake (**A**) and nitric oxide release (**B**) in murine macrophages RAW 264.7 after lipopolysaccharide (LPS) exposure. Cells were pre-treated (24 h) and stimulated with LPS (24 h). Data are reported as the mean ± SEM (*n* = 9). °°° *p* < 0.001, significant difference with respect to Ctrl. *** *p* < 0.001, significant difference with respect to LPS. Statistical analysis performed using ANOVA followed by Dunnett’s Multiple Comparison Post-Test.

The immunomodulatory properties of the extracts and their mixtures were evaluated in murine macrophages RAW 264.7 in terms of the ability to improve the neutral red uptake (Figure 6), which may be representative of the phagocytic capacity, and to protect the cells from the oxidative and inflammatory damage caused by tBOOH (Figure 7 and Figure 8) and LPS (Figure 9). To this end, the cell viability, intracellular ROS levels, apoptosis rate, neutral red uptake and nitric oxide release were measured. Two exposure protocols, i.e., 24 h and 2 h followed by a further 24 h exposure (Figure 1A,B), were applied. 

The ECP extract was able to enhance the neutral red uptake, especially at a lower concentration, inducing about a 1.3- and 2-fold increase after 24 h and 2 h of exposure followed by a second 24 h treatment, respectively (Figure 6A,B). Similarly, the HOP extract induced about a 2-fold increase in the neutral red uptake after both time exposures (Figure 6A,B). The ECP/HOP mixture (70/140 µg/mL) raised the neutral red uptake from 3- to almost 4-fold after 24 h and 2 h plus another 24 h treatment, respectively, thus improving the immunomodulatory effects of the single extracts; conversely, the 140/140 µg/mL mixture achieved an increase like the ECP and HOP extracts (Figure 6A,B).

The cytoprotective properties of the extracts and their mixtures against the pro-oxidant damage of tBOOH in RAW 264.7 murine macrophages were evaluated as previously described (Figure 1C). Despite almost a 4.5-fold reduction induced by tBOOH, the 140/140 µg/mL ECP/HOP mixture almost completely restored the cell viability of murine macrophages, also improving the cytoprotective effects of the single ECP and HOP extracts (Figure 7A). Conversely, the cytoprotection induced by the 70/140 µg/mL ECP/HOP mixture was slightly improved only with respect to ECP; indeed, the cell viability was increased by 1.5-, 1.3- and 2.7-fold by the 70/140 µg/mL ECP/HOP mixture, 70 µg/mL ECP and 140 µg/mL HOP, respectively (Figure 7A). Accordingly, the intracellular ROS levels induced by the pro-oxidant agent tBOOH resulted in an increase by almost 3-fold with respect to the control, while ECP and HOP extracts, as well as their mixtures, counteracted the tBOOH effect from 1.2- to at least 2-fold (Figure 7B). The HOP extract and the ECP/HOP mixtures were especially effective in reducing the ROS levels induced by tBOOH, although without a significant potentiation of the mixtures with respect to the HOP extract (Figure 7B).

Under the same exposure protocol (Figure 1C), tBOOH was also able to affect the proliferation of RAW 264.7 cells, inducing a 32% reduction with respect to the control; moreover, it increased the apoptotic rate by almost 2-fold (Figure 8A,B). All treatments counteracted the tBOOH damage, enhancing the cell proliferation by about 1.3-fold with respect to tBOOH without a significant potentiation of the mixtures with respect to the single extracts (Figure 8A). Similarly, the 140/140 µg/mL ECP/HOP mixture and the ECP extract markedly lowered the apoptosis rate induced by tBOOH, completely restoring the basal levels; conversely, the 70/140 µg/mL ECP/HOP mixture and the HOP extract exhibited weak or null antiapoptotic effects (Figure 8B). 

Given the higher protective properties of 140/140 µg/mL ECP/HOP against the oxidative damage of tBOOH, this mixture was also assessed for its anti-inflammatory effects under stimulation with lypopolisaccharide (LPS) according to the exposure protocol displayed in Figure 1D. At the end of the experiment, the neutral red uptake and the release of nitric oxide were measured. The exposure to LPS did not affect the neutral red uptake while inducing a doubled release of nitric oxide (Figure 9A,B). All treatments similarly increased the neutral red uptake (about 10% increase) with respect to both the control (Figure 9A) and LPS and lowered the release of nitric oxide by about 1.3-fold (Figure 9B). These findings highlighted the cytoprotective and immunomodulatory effects of the ECP/HOP mixtures in RAW 264.7 murine macrophages, counteracting both the oxidative damage of tBOOH and LPS-induced inflammation.

### 3.5. Antiviral Activity against Influenza Virus PR8/H1N1 Strain in Bronchial Epithelial BEAS-2B Cells and in Lung Adenocarcinoma A549 Cells 

The antiviral activity was investigated in both BEAS-2B and A549 cells infected with the influenza virus PR8/H1N1 strain. The cells were subjected to a before, during and post-infection treatment protocol (b.d.p.i.) characterized by a 1 h pre-treatment with the extract and their mixtures, followed by a 1 h co-treatment during the adsorption period and a further 24 h exposure post-infection. The interference of the treatments with the viral replication was evaluated using the hemagglutination assay and the In-Cell Western assay, which provide information about viral titre and infectivity, respectively. Furthermore, the expression of viral proteins and the antioxidant factor Nrf2 was measured. 

**Figure 10:**
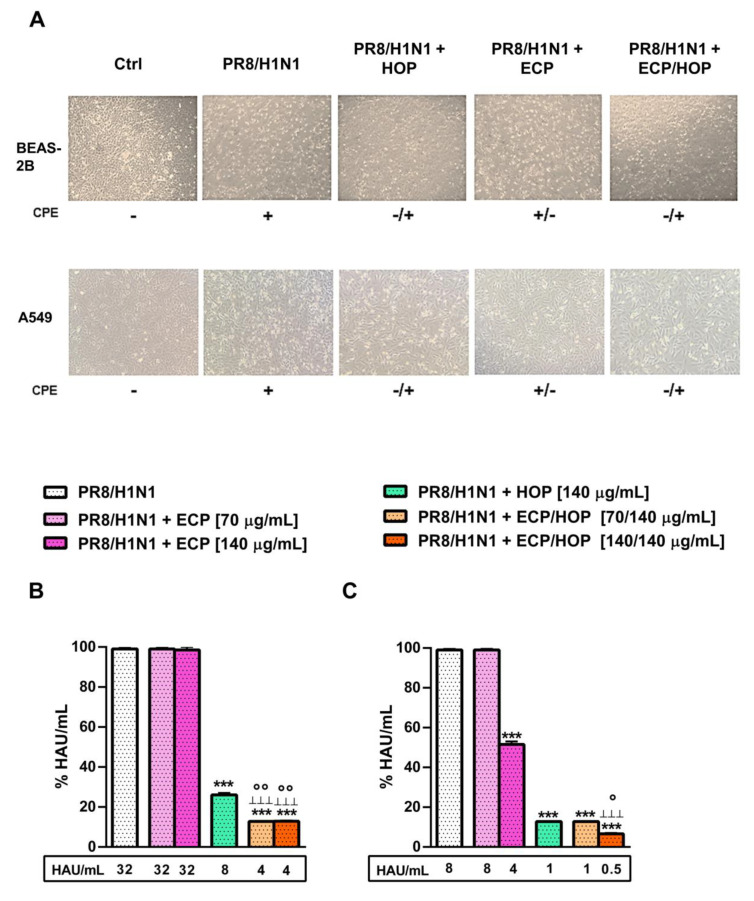
Effect of ECP (70 and 140 µg/mL), HOP (140 µg/mL) and their mixtures (70/140 and 140/140 µg/mL) in bronchial epithelial BEAS-2B cells and lung adenocarcinoma A549 cells infected by influenza virus PR8/H1N1 strain. (**A**) Morphological changes, expressed as cytopathic effect (CPE), induced by 140 µg/mL ECP and HOP extracts and by their 140/140 µg/mL mixture. (**B**,**C**) Viral titre, expressed as units of hemagglution (HAU), in BEAS-2B cells and A549 cells. Cells were treated before the infection (1 h), during the infection with influenza virus PR8/H1N1 strain (1 h) and after the infection (24 h). Data are reported as the mean ± SEM (*n* = 9). *** *p* < 0.001, significant difference with respect to Ctrl. ° *p* < 0.05 and °° *p* < 0.01, significant difference in the ECP/HOP mixture with respect to HOP extract. ^ꞱꞱꞱ^ *p* < 0.00, significant difference in the ECP/HOP mixture with respect to ECP extract. Statistical analysis performed using ANOVA followed by Dunnett’s Multiple Comparison Post-Test.

Under our experimental conditions, the influenza virus PR8/H1N1 strain induced cytopathic effects in both BEAS-2B and A549 cells, which was significantly decreased by the treatments, especially by the HOP and ECP/HOP mixture (Figure 10A). 

The integrity of the cellular monolayer was markedly lowered by infection in both BEAS-2B and A549 cells, while the HOP and ECP/HOP mixtures restored normal morphology (Figure 10A). The mixtures also induced a marked decrease in the viral titre by almost 90% in both BEAS-2B (Figure 10B) and A549 cells (Figure 10C), with an increased effect of the 140/140 µg/mL ECP/HOP mixture with respect to the HOP extract (Figure 10B,C). Based on these results, the subsequent antiviral studies were focused on the 140/140 µg/mL ECP/HOP mixture in comparison with the corresponding single extracts. 

**Figure 11:**
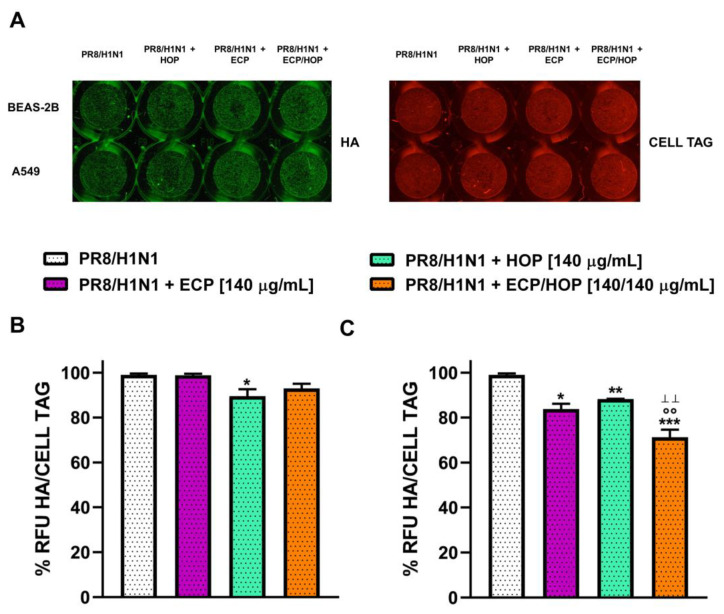
Effect of ECP (140 µg/mL), HOP (140 µg/mL) and their mixture (140/140 µg/mL) on the infective capacity of the supernatants of bronchial epithelial BEAS-2B cells and lung adenocarcinoma A549 cells. Cells were treated before the infection (1 h), during the infection with influenza virus PR8/H1N1 strain (1 h) and post-infection (24 h). (**A**) In Cell Western assay (ICW) of Hemagglutinin (HA) expression in infected BEAS-2B and A549 cells. The green fluorescence is representative of viral protein expression and red fluorescence of Cell Tag staining of cell monolayer. (**B**,**C**) Percentage (%) of relative fluorescence units (RFU) of HA protein expression normalized to Cell Tag in BEAS-2B and A549 cells, respectively. Data are reported as the mean ± SEM (*n* = 9). * *p* < 0.05, ** *p* < 0.01 and *** *p* < 0.001, significant difference with respect to the infected control. °° *p* < 0.01, significant difference in the ECP/HOP mixture with respect to HOP extract. ^ꞱꞱ^ *p* < 0.01, significant difference in the ECP/HOP mixture with respect to ECP extract at the corresponding concentration. Statistical analysis performed using ANOVA followed by Dunnett’s Multiple Comparison Post-Test.

The 140/140 µg/mL ECP/HOP mixture was evaluated also for its capacity to interfere with the viral replication through the In-Cell Western assay, which provided insights into the infective potential of cell supernatants collected after exposure to the infection and treatments. To this end, the supernatants of BEAS-2B and A549 cells, previously treated with the tested sample under the b.d.p.i protocol, were collected and used to infect canine kidney MDCK cells, which are highly permissive to influenza virus replication, as previously reported [8], and the hemagglutinin expression was determined. Despite the results obtained against the first infection by the influenza virus PR8/H1N1 strain, the extracts and their mixture only slightly affected the residual infective capacity of the BEAS-2B supernatants, with a moderate reduction against that of the A549 ones (Figure 11A,B). In the last case, the ECP/HOP mixture lowered the infective capacity of A549 supernatants by 1.4-fold, with a significantly increased effect with respect to the extracts (Figure 11B).

**Figure 12:**
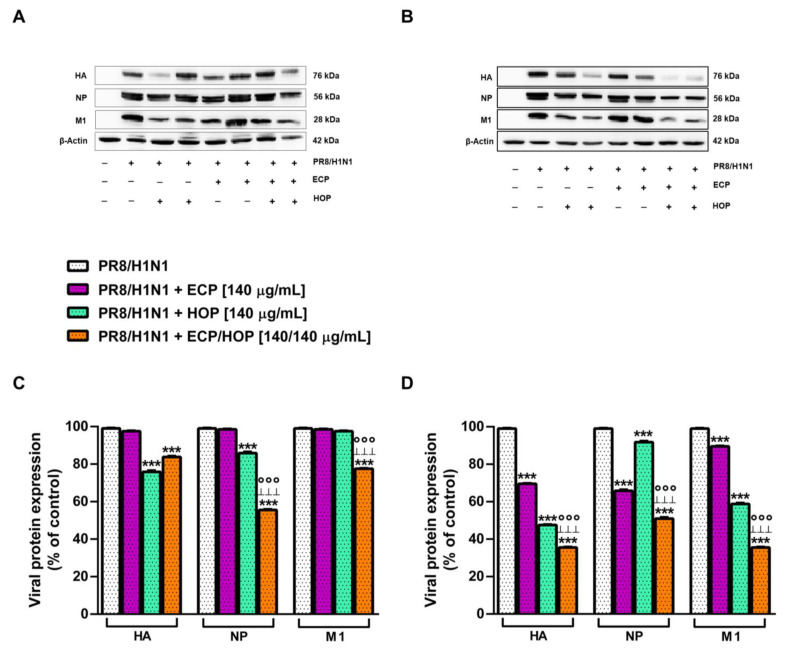
Effect of ECP (140 µg/mL), HOP (140 µg/mL) and their mixture (140/140 µg/mL) on the expression of viral proteins hemagglutinin (HA), nucleoprotein (NP) and matrix protein (M1) in bronchial epithelial BEAS-2B cells and lung adenocarcinoma A549 cells. Cells were treated before the infection (1 h), during the infection with influenza virus PR8/H1N1 strain (1 h) and after the infection (24 h). (**A**,**B**) Representative Western blotting membranes displaying the viral proteins and β -actin (protein loading control) in BEAS-2B and A549 cells, respectively. (**C**,**D**). Bar graphs of the viral protein densitometric analysis in BEAS-2B and A549 cells, respectively (data expressed as mean ± standard error obtained from at least three independent experiments). *** *p* < 0.001, significant difference with respect to the infected control. °°° *p* < 0.001, significant difference in the ECP/HOP mixture with respect to HOP extract. ^ꞱꞱꞱ^ *p* < 0.001, significant difference in the ECP/HOP mixture with respect to ECP extract. Statistical analysis performed using ANOVA followed by Dunnett’s Multiple Comparison Post-Test.

In order to investigate the steps of viral replication affected by the treatments, the expression of the viral proteins hemagglutinin (HA), nucleoprotein (NP) and matrix protein (M1), synthesized at different steps of influenza virus replication, was measured in BEAS-2B and A549 cells. Indeed, NP is an early viral protein, while HA and M1 proteins are synthesized in the late step of viral replication [49]. In BEAS-2B cells, ECP extract did not affect the expression of these viral proteins, despite a significant reduction induced by the HOP extract and ECP/HOP mixture (Figure 12A,C): HA and NP were reduced by about 24% and 14% by HOP and by 16% and 45% by the ECP/HOP mixture, respectively. Moreover, the HOP extract did not affect the M1 protein expression, despite a 23% reduction induced by the ECP/HOP mixture (Figure 12C). There were more potent antiviral properties in A549 cells compared to BEAS-2B cells, and the mixture exhibited greater inhibition of viral protein expression than the individual extracts (Figure 12B,D). Indeed, HA, NP and M1 proteins were reduced by ECP, HOP and their mixture by 30%, 53% and 65%, by 30%, 10% and 49% and by 10%, 45% and 65%, respectively (Figure 12D). The original Western blotting membranes are reported in Figures S1 and S2.

**Figure 13:**
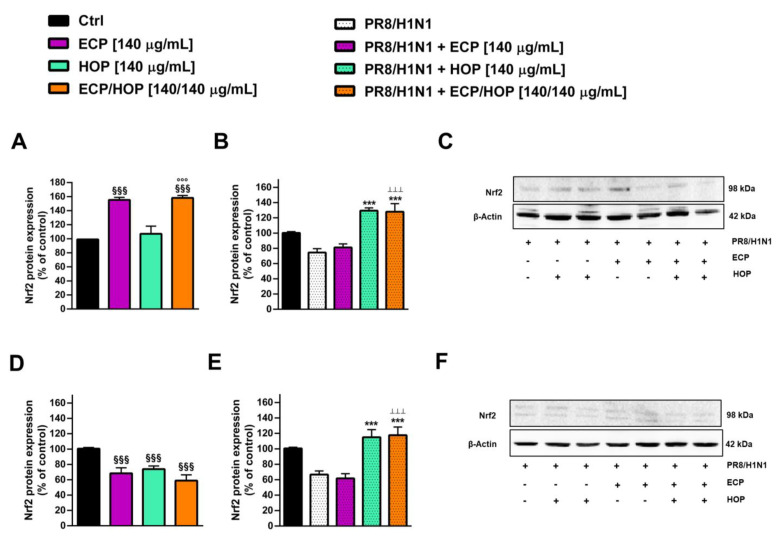
Effect of ECP (140 µg/mL), HOP (140 µg/mL) and their mixture (140/140 µg/mL) on the expression of Nrf2 in bronchial epithelial BEAS-2B cells (**A**–**C**) and lung adenocarcinoma A549 cells (**D**–**F**). Cells were treated before the infection (1 h), during the infection with influenza virus PR8/H1N1 strain (1 h) and after the infection (24 h). The bar graphs represent the Nrf2 densitometric analysis in BEAS-2B (**A**,**B**) and A549 (**D**,**E**) cells (data expressed as mean ± standard error obtained from at least three independent experiments). ^§§§^ *p* < 0.001, significant difference with respect to control. *** *p* < 0.001, significant difference with respect to the infected control. °°° *p* < 0.001, significant difference in the ECP/HOP mixture with respect to HOP extract. ^ꞱꞱꞱ^ *p* < 0.001, significant difference in the ECP/HOP mixture with respect to ECP extract. Statistical analysis performed using ANOVA followed by Dunnett’s Multiple Comparison Post-Test. (**C**,**F**) Western blotting membranes displaying Nrf2 and β -actin (protein loading control) in BEAS-2B and A549 cells.

In order to evaluate the possible mechanisms underlying the antiviral properties of the ECP/HOP mixture and taking into account the ability of influenza infection to impair the redox state of the host cells, we assessed the ability of the treatment to modulate the Nrf2 expression in BEAS-2B and A549 cells under the b.d.p.i. protocol (Figure 13). 

In BEAS-2B cells, the basal levels of Nrf2 were markedly enhanced (by about 1.6-fold) by ECP and ECP/HOP treatments, suggesting the stimulation of the host antioxidant defences and thus a better capability of the host cell to counteract the oxidative damage (Figure 13A). Moreover, the ECP/HOP mixture produced a slight (by 1.02-fold) but not significant increase in the Nrf2 expression with respect to the ECP extract (Figure 13A). In infected BEAS-2B cells, the Nrf2 expression was decreased by 1.4-fold by the influenza virus PR8/H1N1 strain, while both the HOP extracts and ECP/HOP mixture markedly increased the Nrf2 expression with respect to the infected control (about 1.7-fold increase) (Figure 13B,C). In A549 cells, both the extracts and their mixture lowered the basal expression of Nrf2 by about 1.4-fold, thus highlighting the different behaviour in normal and cancerous cells (Figure 13D). 

Considering the upregulation of Nrf2 in A549 cells, which is known to make the cells highly resistant to oxidative stress and to pharmacological treatments, the lowered basal expression induced by the extracts and their mixture suggest a possible control on this altered pathway, although the true role and the mechanisms involved remain to be clarified. The PR8/H1N1 virus infection led to a 1.5-fold lowering of Nrf2 expression, which was counteracted by the HOP extract and ECP/HOP without the effects of ECP (Figure 13E,F): indeed, about a 1.2-fold increase in Nrf2 expression with respect to the levels of the infected control was achieved. The original Western blotting membranes are reported in Figures S3 and S4. 

## 4. Discussion and Conclusions

The health emergency associated with respiratory viral infections, exemplified by the coronavirus pandemic, underscores the increasing necessity to devise innovative preventive strategies aimed at reducing the incidence of upper respiratory tract viral diseases and their associated complications. These strategies are of utmost importance, especially for vulnerable subjects, in which viral infections can have devastating consequences, resulting in serious health complications, hospitalizations and even fatalities [50]. Therefore, implementing preventive measures and developing effective treatments may lower the burden of respiratory viral infections and enhance overall public health. 

Our previous findings highlighted a promising antiviral activity of a hydroalcoholic extract from the hop inflorescences (named the HOP extract) against PR8/H1N1 virus infection, likely associated with its antioxidant power, thus strengthening our interest in this product as a possible preventive or adjuvant strategy to fight respiratory viral diseases [26]. In the present study, we developed a novel formulation by blending a HOP extract with a hydroalcoholic extract from echinacea (named ECP) to harness their collective properties, thereby improving the protective capabilities against respiratory viruses. Indeed, both hop (*Humulus lupulus* L.) and echinacea (*Echinacea purpurea* (L.) Moench) exhibited promising antioxidant, antiviral and anti-inflammatory activities that make them interesting candidates for the prevention and treatment of upper respiratory tract infections [24,29]. Moreover, echinacea is known to possess immunostimulating properties, mainly harnessed to relieve the common cold and upper respiratory tract ailments [29,51].

The developed ECP/HOP mixture (named ECHOPvir) was investigated for its in vitro cytoprotective, immunomodulatory and antiviral properties; moreover, the phenolic composition of the extracts was determined in order to chemically characterize the tested samples and to identify the potential bioactive compounds.

The obtained results highlighted the ability of the extracts and their mixtures to counteract the tBOOH-induced oxidative damage and to lower the intracellular ROS levels in both BEAS-2B and A549 airway cell models. Moreover, they were able to restore the apoptotic basal level impaired by tBOOH. The induced oxidative stress was more pronounced in BEAS-2B cells compared to A549 cells, as the latter showed lower susceptibility to oxidative stress, likely due to a physiological redox imbalance. 

The ECP/HOP 140/140 µg/mL mixture usually exhibited significantly increased cytoprotective effects with respect to the extracts alone, thus suggesting that synergistic or additive effects among the phytochemicals in the mixture may occur. These findings can be explained on the basis of the known antioxidant activities of both HOP and ECP extracts, previously highlighted in different experimental models [26,51,52,53] and also confirmed by the lowering of the intracellular ROS levels in our cell models. Antioxidant properties were also reported for the prenylflavonoid xanthohumol, the bitter acids humulone and lupulone and for other phenolic compounds from hop cones [19,22,54]. Likewise, echinacea showed significant antioxidant properties in numerous studies: polyphenols, including chicoric acid, which is a typical constituent of *Echinacea* spp. [55], fructans and alkylamides, were considered the main bioactive compounds [29,56,57]. 

In line with our results, other studies highlighted the protective properties of hop cone extracts towards the oxidative stress induced by diverse stimuli (e.g., iron overload) both in in vitro and in vivo models [58,59]. Moreover, xanthohumol exhibited protective properties in different experimental models of brain and liver injury, which were associated with its antioxidant power [58,60,61,62,63]. 

In our previous study, different mechanisms of both direct and indirect antioxidant activity were highlighted for the HOP extract; particularly, it showed the ability to inhibit the lipid peroxidation [26], thus suggesting that this can represent a mechanism by which HOP counteracts the tBOOH oxidative damage on the cell membrane and lowers the generation of reactive oxygen species, leading to cytoprotective effects. 

In the present study, ECP extracts were found to be great sources of chicoric acid, caftaric acid and 3-hydroxytyrosol and HOP extracts of 2,3-dimethylbenzoic acid, thymol and isoquercetin. Scientific evidence strengthened in recent years the potential health benefits of these compounds, mainly ascribed to their antioxidant power [64,65,66,67,68,69,70]. Accordingly, phenolic acids, such as t-cinnamic, p-coumaric, benzoic, caffeic, ferulic, rosmarinic and syringic acids, identified in the ECP and HOP extracts, were highlighted to possess promising healing properties [71], which suggest their possible contribution to the antioxidant and cytoprotective effects of the ECP/HOP mixture. 

These findings suggest a possible involvement of whole phytocomplexes in the cytoprotection by ECP/HOP against the oxidative damage of tBOOH. The increased effect of the mixture with respect to each extract allows us to hypothesize that synergistic or additive interactions among the phytochemicals of ECP and HOP can occur, but more in-depth future studies are needed to clarify this issue.

Considering the greater susceptibility of BEAS-2B cells to oxidative damage, we also evaluated the effect of the tested extract and their mixtures on the apoptosis rate in comparison with the cell proliferation abilities under the oxidative stress induced by tBOOH. 

Scientific evidence revealed that apoptosis is activated in response to oxidative stress [72,73,74]; however, chronic exposure to pro-oxidant agents may impair this mechanism of programmed cell death, thus likely favouring the survival and further degeneration of damaged cells [75]. In this respect, Shim et al. [76] reported that cells stimulate apoptotic processes as a defense mechanism to prevent viral replication, while the virus tends to inhibit this mechanism. Under our experimental conditions, which mime an acute exposure to a pro-oxidant agent, we found that tBOOH significantly increased the apoptosis rate while lowering cell proliferation. Conversely, both ECP and HOP extracts, as well as their mixtures, counteracted the tBOOH effects and almost restored normal proliferation, also blocking apoptotic cell death. These findings further support the cytoprotective power of the ECP/HOP mixture. 

Under our experimental conditions, we also found a lower susceptibility of A549 cells to the oxidative damage of tBOOH than BEAS-2B cells; indeed, the intracellular ROS levels were only weakly affected by the pro-oxidant treatment. The A549 resilience towards the tBOOH-induced oxidative stress may arise from the upregulation of the antioxidant cell defences typical of this cell line [47]. Indeed, A549 cells are Nrf2 (NF-E2-related factor 2)-addicted cancer cells, as they carry a constitutive Nrf2 activation, which is a master regulator of several cytoprotective genes and underpins their proliferative abilities [77]. Nrf2 knockdown by siRNA significantly impaired the proliferative abilities of A549 cells, thus suggesting that it can represent a suitable strategy against Nrf2-addicted cancers [78]. 

Nonetheless, the reduction in ROS production, indicating protection from oxidative damage, was evident and statistically significant in both cell models under all conditions and treatment times, suggesting a possible role of the EPC and HOP extracts as well as of their mixtures as both antioxidant and cytoprotective agents and regulators of Nrf2-induced cancer resistance. In line with our hypothesis, a regulatory effect by hop cone extracts and xanthohumol on the Nrf2 cascade has been reported in other in vitro and in vivo studies [58,60,61,62,63,79].

Under our experimental conditions, we also highlighted interesting immunomodulatory properties for ECP and HOP extracts, as well as for their mixtures, in RAW 264.7 murine macrophages. They were able to increase both the basal and LPS-induced neutral red uptake of the RAW 264.7 cells, prevent the oxidative damage of tBOOH and partly limit the release of proinflammatory factors. This evidence suggests a possibility for the developed mixture to boost the immune system and of improving its activation to counteract exogenous injuries, such as viral infections. Along with these properties, the tested samples, especially the HOP extract and the ECP/HOP mixtures, also exhibited antioxidant properties. These findings confirm our hypothesis that the cytoprotective activity of ECP/HOP is closely related to the antioxidant activity of the extracts, mainly HOP, according to previous studies [26]. 

The immunostimulating properties of echinacea are well known [51] and have been ascribed mainly to glycoproteins, alkylamides and polysaccharides. Particularly, alkylamides are known to activate the CB2 cannabinoid receptors, involved in the immune response, resulting in an increased production of anti-inflammatory factors, such as interleukin (IL)-10, and the inhibition of inflammatory ones (e.g., IL-1, IL-6 TNF-α, TNF-β and nitric oxide) [28]. Similarly, hop has been reported to possess anti-inflammatory properties, owing to its ability to inhibit pro-inflammatory cytokine release [18] and the NF-kB cascade [57]. A terpene-enriched extract from *H. lupulus* produced anti-inflammatory effects and potentiated those of cannabidiol in LPS-activated RAW 264.7 murine macrophages [80]. Among the hop phytochemicals, xanthohumol has been reported to cause a marked reduction in nitric oxide (NO) synthesis through the inhibition of nitric oxide synthase (iNOS) [18]; it also blocked the production of inflammatory mediators in LPS- and IFN-γ-activated macrophages by suppressing the activation of NF-κB, STAT-1α and IRF-1 cascades [81]. Furthermore, several polyphenols detected in the ECP and HOP extracts, including chicoric acid, caffeic acid, ferulic acid and benzoic acid derivatives, as well as resveratrol, catechin and thymol, have been reported to act as immune system modulators, owing to their antioxidant power [82,83,84,85,86,87]. 

Based on this evidence, the ECP/HOP blend emerges as a rich phytocomplex in which several phytochemicals cooperate to provide marked antioxidant, anti-inflammatory and immunomodulatory properties, suggesting its promising potential in the battle against viral infections and their complications.

In this respect, our results highlighted the antiviral abilities of the ECP/HOP mixture against influenza PR8/H1N1 virus infection in both BEAS-2B and A549 cell models. These effects were mainly associated with the presence of the HOP extract, although increased by the mixture with respect to the extract alone. Both the HOP extract and the ECP/HOP mixture were able to inhibit the viral replication and affect the expression of the viral proteins hemagglutinin (HA), nucleoprotein (NP) and matrix protein (M1). Among them, NP plays an important role in regulating viral genome transcription and replication, as it binds the viral RNA segments and viral polymerase to form viral ribonucleoproteins (vRNAs) [88], while M1 facilitates the entry of the virus into the host cell, and it is synthesized in the late step of viral replication [89]. 

Based on our findings, the ECP/HOP mixture demonstrated the ability to inhibit various stages of PR8/H1N1 viral infection, including viral entry into host cells, genome transcription and replication. This suggests its potential as a preventive strategy against viral diseases. 

Previous evidence has shown that both echinacea and hop phytocomplexes possess antiviral properties, suggesting their joint effect on the outcomes of the ECP/HOP blend. Echinacea exhibited virucidal properties against enveloped viruses, such as influenza and coronavirus [32,90,91]. Particularly, a hydroethanolic extract (65% *v*/*v*) from the freshly harvested aerial parts and root of *E. purpurea* (95% and 5%, respectively), standardized to contain 5 mg/100 g of dodecatetraenoic acid isobutylamide, was found to be effective against a broad number of coronaviruses, including the common cold and the highly pathogenic SARS-CoV-2 strains [92]. These benefits have been also confirmed in randomized clinical trials carried out in adults and children [93,94,95]; however, further clinical confirmations are required to support the use of this Echinacea extract against viral infections [96]. Interestingly, some studies showed chicoric acid, a key phenolic compound from Echinacea aerial parts and roots, to interfere with SARS-CoV-2 proteins, suggesting that it can represent one of the possible bioactive compounds [97,98]. Accordingly, a previous meta-analysis highlighted that within 2 to 4 months of the preventive use of Echinacea, the risk of pneumonia secondary to viral respiratory infections was significantly lowered [99]. 

Along with Echinacea, hop cones were also reported to possess antiviral properties [26,100,101]. Recently, Bouback et al. showed that a boiled water extract from the dried whole plant of *H. lupulus* possessed anticoronaviral properties against both MERS-CoV and SARS-CoV-2, blocking the viral ability to infect and replicate inside the host cells: these effects are mainly ascribed to its polyphenolic compounds [102]. The authors also predicted in silico that the compounds 5′-prenylxanthohumol, dehydrocycloxanthohumol hydrate, isoxanthohumol, xanthogalenol, 6-prenylnaringenin, catechin gallate, epicatechin gallate, 8-prenylnaringenin and xanthohumol may likely act as SARS-CoV-2 and MERS-CoV inhibitors by binding to the receptor-binding domain (RBD) of the spike proteins [102]. According to these predictions, previous studies have focused on xanthohumol as a broad-spectrum antiviral agent [103,104,105,106]. Lin et al. [107] also highlighted that xanthohumol was a potent pan-inhibitor for various coronaviruses, acting as a protease inhibitor. However, other compounds can contribute to the antiviral effects of the ECP/HOP mixture. Indeed, the bitter acids from hop seem to interact with the virus envelope, altering its permeability and structure [26], while tannins can inactivate the viral protein, as previously highlighted [33]; moreover, antiviral activities have been reported for isoquercetin, ferulic acid and hydroxytyrosol as well [108,109,110,111]. 

In an attempt to clarify the possible mechanisms underlying the antiviral effects of the ECP/HOP mixture and taking into account the crucial role of oxidative stress in the host cell in favouring viral replication and infection development [8], we also studied the ability of the mixture to directly stimulate cytoprotective mechanisms in the host cell through the regulation of the Nrf2 pathway. 

Nrf2 is a transcription factor involved in the antioxidant response through the regulation of NADPH regeneration and the expression of detoxifying enzymes, like heme oxygenase-1 (HO-1) and glutathione-S-transferase (GST), anti-inflammatory and antioxidant proteins and cytoprotective genes. Under normal conditions, Nrf2 is bound to Keap-1, which induces its rapid degradation, thus maintaining low intracellular levels. Under oxidative stress conditions (like viral infections and inflammation), Keap-1 becomes oxidized, leading to the release of Nrf2, which may translocate into the nucleus to promote the expression of cytoprotective and antioxidant factors [112,113]. 

Interestingly, the HOP extract and the mixture markedly enhanced the Nrf2 expression, impaired by the PR8/H1N1 virus infection, in both BEAS-2B and A549 cell models. In BEAS cells, an increased basal expression of Nrf2 was found as well. These effects agree with previous evidence, highlighting that the HOP extract was able to increase the GSH levels, thus likely counteracting the intracellular oxidative stress [26]. Previous evidence also highlighted that some key compounds detected in the ECP and HOP extracts, such as chicoric acid, xanthohumol, hydroxytyrosol, thymol and isoquercetin, act as Nrf2 modulators [79,114,115,116,117,118]. 

Altogether, this evidence strengthens our interest in the ECO/HOP blend as a source of diverse powerful bioactive agents, endowed with cytoprotective, antioxidant, immunostimulating and antiviral activities, that can be harnessed against upper respiratory viral infections. Despite these promising results, some limitations of our study warrant consideration. The first limitation concerns the reliability of in vitro models in predicting in vivo outcomes. Indeed, the in vitro models employed in this study did not allow us to account for the fate of the ECP and HOP extracts and their mixture within the body, such as how they are absorbed, metabolized, distributed and eliminated: these factors may significantly affect the efficacy and safety of the treatment. Additionally, the controlled laboratory conditions do not accurately simulate the complexities of viral infections and the possible development of resistance within living organisms. Consequently, future in vivo studies and clinical investigations are imperative for obtaining a more comprehensive understanding of the efficacy and safety of the ECP/HOP blend in respiratory infections and of the mechanisms of action. A further issue to be considered concerns the standardization of the ECP/HOP blend. Indeed, although in the present study, the ECP and HOP extracts were produced through standardized methodologies and certified conditions, the intrinsic variability of herbal drugs, owing to diverse natural factors [119], may lead to differences in the chemical composition across different batches of the same extracts, which in turn can affect the product consistency, efficacy and safety. Controlling this chemical variability is essential for allowing the future nutraceutical development of the ECP/HOP blend. In this context, identifying the bioactive compounds and ensuring their effective amount in the mixture, along with optimizing the studied mixture with respect to specific bioactive groups of compounds, represent a possible strategy to address this challenge.

In conclusion, our results unveil a wide array of cytoprotective, antioxidant, anti-inflammatory, immunostimulatory and antiviral properties for the ECP/HOP blend, which appears promising for the prevention and treatment of respiratory viral diseases. The mixture may serve as both a preventive and adjuvant strategy not only directly interfering with viral replication but also enhancing endogenous antioxidant and immune defenses, thereby bolstering the body’s resilience against viral threats. 

The present findings encourage further research to comprehensively characterize the mechanisms underpinning the benefits of the ECP/HOP blend and the involved bioactive compounds and to confirm its efficacy and safety in vivo. Moreover, exploring optimized extracts and assessing the impact of digestive processes on the bioactivities of the ECP/HOP mixture could further enhance its appeal and bolster its future nutraceutical applications. 

## Figures and Tables

**Table 1:** Amount of total polyphenols, tannins and flavonoids in HOP and ECP extracts (*n* = 3).

Sample	Total Polyphenols	Tannins	Flavonoids
	µg TAE/mg of the Sample (Mean ± SE)		µg QE/mg of the Sample (Mean ± SE)
ECP	4.89 ± 0.40	0.78 ± 0.01	3.11 ± 0.72
HOP	7.11 ± 0.35 ***	1.72 ± 0.06 ***	3.81 ± 0.59

TAE, tannic acid equivalents; QE, quercetin equivalents. *** *p* < 0.001 vs. ECP (t-Student’s test).

**Table 2:** Phenolic composition of HOP and ECP extracts according to HPLC-PDA analysis.

Compounds	ECP	HOP
	µg/mg of the Sample (Mean ± SD)	
Benzoic acid	0.03 ± 0.01	0.41 ± 0.03
Caffeic acid	0.39 ± 0.02	0.06 ± 0.01
Caftaric acid	1.47 ± 0.03	0.16 ± 0.02
Carvacrol	nd	nd
Catechin	0.34 ± 0.03	0.52 ± 0.02
Chicoric acid	7.76 ± 0.23	nd
Chlorogenic acid	0.24 ± 0.01	0.06 ± 0.02
t-Cinnamic acid	0.01 ± 0.002	1.04 ± 0.06
p-Coumaric acid	nd	0.51 ± 0.03
2,3-Dimethylbenzoic acid	nd	4.43 ± 0.23
Epicatechin	0.57 ± 0.03	BLD
t-Ferulic acid	0.25 ± 0.02	0.22 ± 0.03
Gallic acid	0.28 ± 0.01	0.25 ± 0.01
Kaempferol	nd	0.32 ± 0.06
Hesperetin	0.02 ± 0.003	nd
4-Hydroxybenzoic acid	nd	0.08 ± 0.02
3-Hydroxytyrosol	2.20 ± 0.11	0.61 ± 0.01
Hyperoside	nd	nd
Isoquercetin	nd	1.61 ± 0.06
Loganic acid	nd	nd
Naringenin	nd	nd
Quercetin	BLD	nd
Resveratrol	nd	0.06 ± 0.01
Rosmarinic acid	nd	0.12 ± 0.02
Rutin	nd	nd
Thymol	nd	4.09 ± 0.11
Syringic acid	0.16 ± 0.02	nd
Syringaldehyde	nd	0.07 ± 0.01
Vanillic acid	nd	nd

nd, not detected; BLD, below the limit of detection.

## Data Availability

Not applicable.

## Supplementary Materials

The following supporting information can be downloaded at: https://www.mdpi.com/article/10.3390/nu15204380/s1, Figure S1: Original Western blotting membrane used to evaluate the expression of the viral proteins (A) and of β-actin (protein loading control) in bronchial epithelial BEAS-2B cells. HA, hemagglutinin; NP, nucleoprotein; and M1, matrix protein. (1) Control; (2) PR8/H1N1; (3) PR8/H1N1 + HOP 140 μg/mL; (4) PR8/H1N1 + ECP 140 μg/mL; and (5) PR8/H1N1 + ECP/HOP 140/140 μg/mL. Figure S2: Original Western blotting membrane used to evaluate the expression of the viral proteins (A) and of β-actin (protein loading control) in lung adenocarcinoma A549 cells. HA, hemagglutinin; NP, nucleoprotein; M1, matrix protein. (1) Control; (2) PR8/H1N1; (3) PR8/H1N1 + HOP 140 μg/mL; (4) PR8/H1N1 + ECP 140 μg/mL; and (5) PR8/H1N1 + ECP/HOP 140/140 μg/mL. Figure S3: Original Western blotting membrane used to evaluate the expression of Nrf2 (A) and of β-actin (protein loading control) in bronchial epithelial BEAS-2B cells. (1) PR8/H1N1; (2) PR8/H1N1 + HOP 140 μg/mL; (3) PR8/H1N1 + ECP 140 μg/mL; and (4) PR8/H1N1 + ECP/HOP 140/140 μg/mL. Figure S4: Original Western blotting membrane used to evaluate the expression of Nrf2 (A) and of β-actin (protein loading control) in lung adenocarcinoma A549 cells. (1) PR8/H1N1; (2) PR8/H1N1 + HOP 140 μg/mL; (3) PR8/H1N1 + ECP 140 μg/mL; and (4) PR8/H1N1 + ECP/HOP 140/140 μg/mL.
