# Supplementary material for: ECHOPvir: A Mixture of Echinacea and Hop Extracts Endowed with Cytoprotective, Immunomodulatory and Antiviral Properties
Source: Nutrients. 2023 Oct 16;15(20):4380. doi: 10.3390/nu15204380 (PMC10609862; doi:10.3390/nu15204380)
Supplement: Supplementary file 1 [file nutrients-15-04380-s001.zip › nutrients-2638392-supplementary.pdf]

*nutrients*

MDPI

*Supplementary Materials*

# ECHOPvir: A Mixture of Echinacea and Hop Extracts Endowed with Cytoprotective, Immunomodulatory and Antiviral Properties

**Ester Percaccio [1,†], Marta De Angelis [2,3,†], Alessandra Acquaviva [4], Giovanna Nicotra [5], Claudio Ferrante [4], Gabriela Mazzanti [1], Silvia Di Giacomo [1,6], Lucia Nencioni [2,‡] and Antonella Di Sotto [1,*,‡]**

[1] Department of Physiology and Pharmacology "V. Erspamer", Sapienza University of Rome, P.le Aldo Moro 5, 00185 Rome, Italy; ester.percaccio@uniroma1.it (E.P.); gabriela.mazzanti@gmail.com (G.M.)
[2] Laboratory Affiliated to Istituto Pasteur Italia-Fondazione Cenci Bolognetti, Department of Public Health and Infectious Diseases, Sapienza University of Rome, P.le Aldo Moro 5, 00185 Rome, Italy; marta.deangelis@uniroma1.it (M.D.A.); lucia.nencioni@uniroma1.it (L.N.)
[3] Laboratory of Virology, Department of Molecular Medicine, Sapienza University of Rome, 00185 Rome, Italy
[4] Department of Pharmacy, Botanic Garden "Giardino dei Semplici", Università degli Studi "Gabriele d'Annunzio", Via dei Vestini 31, 66100 Chieti, Italy; alessandra.acquaviva@unich.it (A.A.); claudio.ferrante@unich.it (C.F.)
[5] EPO S.r.l., 20141 Milan, Italy; gnicotra@eposrl.com
[6] Unit of Human Nutrition and Health, Department of Food Safety, Nutrition and Veterinary Public Health, National Institute of Health, 00161 Rome, Italy; silvia.digiacomo@iss.it
* Correspondence: antonella.disotto@uniroma1.it
† These authors contributed equally to this work.
‡ These authors contributed equally to this work.

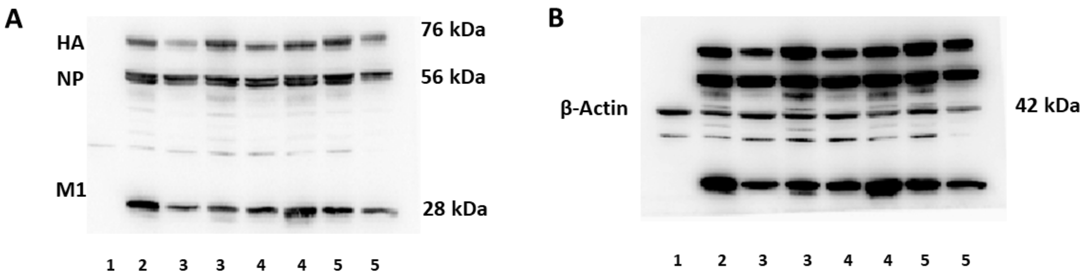


**Figure S1.** Original Western blotting membrane used to evaluate the expression of the viral proteins (A) and of β-actin (protein loading control) in bronchial epithelial BEAS-2B cells. HA, hemagglutinin; NP, nucleoprotein; M1, matrix protein. 1) Control; 2) PR8/H1N1; 3) PR8/H1N1 + HOP 140 μg/mL; 4) PR8/H1N1 + ECP 140 μg/mL; 5) PR8/H1N1 + ECP/HOP 140/140 μg/mL.

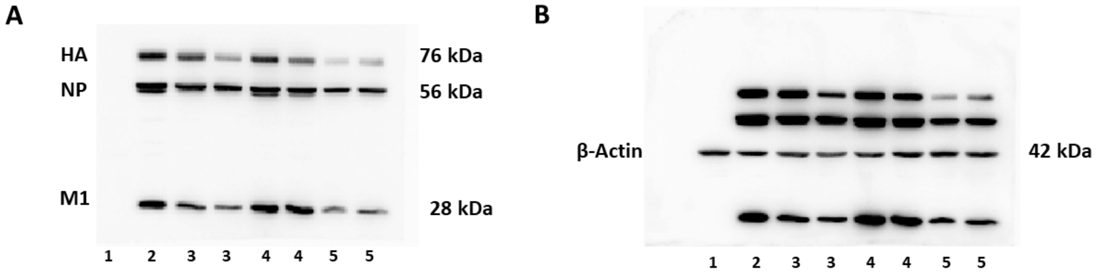

**Figure S2.** Original Western blotting membrane used to evaluate the expression of the viral proteins (A) and of β-actin (protein loading control) in lung adenocarcinoma A549 cells. HA, hemagglutinin; NP, nucleoprotein; M1, matrix protein. 1) Control; 2) PR8/H1N1; 3) PR8/H1N1 + HOP 140 μg/mL; 4) PR8/H1N1 + ECP 140 μg/mL; 5) PR8/H1N1 + ECP/HOP 140/140 μg/mL.

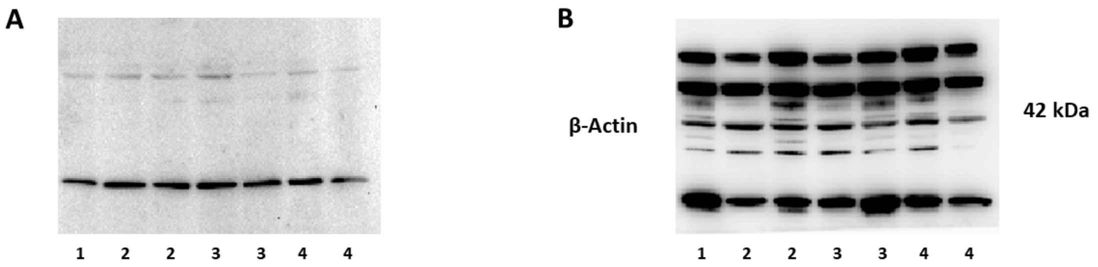


**Figure S3.** Original Western blotting membrane used to evaluate the expression of Nrf2 (A) and of β-actin (protein loading control) in bronchial epithelial BEAS-2B cells. 1) Control; 2) PR8/H1N1; 3) PR8/H1N1 + HOP 140 μg/mL; 4) PR8/H1N1 + ECP 140 μg/mL; 5) PR8/H1N1 + ECP/HOP 140/140 μg/mL.

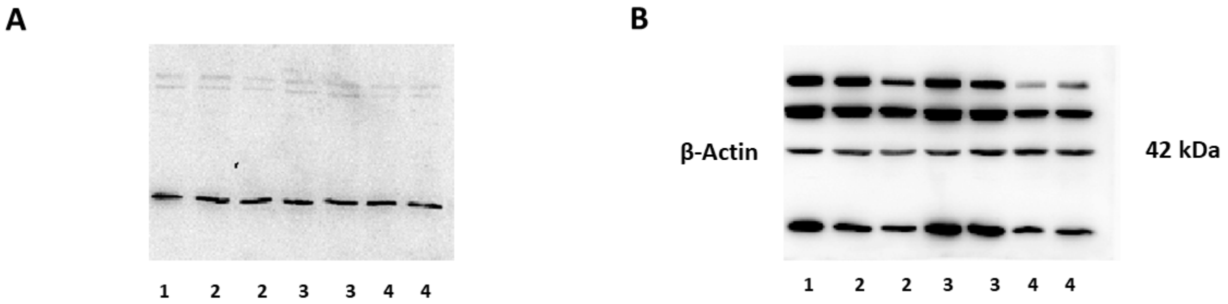


**Figure S4.** Original Western blotting membrane used to evaluate the expression of the Nrf2 (A) and of β-actin (protein loading control) in lung adenocarcinoma A549 cells. HA, hemagglutinin; NP, nucleoprotein; M1, matrix protein. 1) Control; 2) PR8/H1N1; 3) PR8/H1N1 + HOP 140 μg/mL; 4) PR8/H1N1 + ECP 140 μg/mL; 5) PR8/H1N1 + ECP/HOP 140/140 μg/mL.

**Disclaimer/Publisher's Note:** The statements, opinions and data contained in all publications are solely those of the individual author(s) and contributor(s) and not of MDPI and/or the editor(s). MDPI and/or the editor(s) disclaim responsibility for any injury to people or property resulting from any ideas, methods, instructions or products referred to in the content.
